# Anti‐aging pharmacology in cutaneous wound healing: effects of metformin, resveratrol, and rapamycin by local application

**DOI:** 10.1111/acel.12635

**Published:** 2017-07-05

**Authors:** Pan Zhao, Bing‐Dong Sui, Nu Liu, Ya‐Jie Lv, Chen‐Xi Zheng, Yong‐Bo Lu, Wen‐Tao Huang, Cui‐Hong Zhou, Ji Chen, Dan‐Lin Pang, Dong‐Dong Fei, Kun Xuan, Cheng‐Hu Hu, Yan Jin

**Affiliations:** ^1^ State Key Laboratory of Military Stomatology & National Clinical Research Center for Oral Diseases & Shaanxi International Joint Research Center for Oral Diseases Center for Tissue Engineering School of Stomatology Fourth Military Medical University Xi'an Shaanxi 710032 China; ^2^ Xi'an Institute of Tissue Engineering and Regenerative Medicine Xi'an Shaanxi 710032 China; ^3^ Research and Development Center for Tissue Engineering Fourth Military Medical University Xi'an Shaanxi 710032 China; ^4^ Department of Periodontology Stomatological Hospital Zunyi Medical College Zunyi Guizhou 563003 China; ^5^ Department of Dermatology Tangdu Hospital Fourth Military Medical University Xi'an Shaanxi 710069 China

**Keywords:** aged skin, AMPK pathway, anti‐aging pharmacology, metformin, vascularization, wound healing

## Abstract

Cutaneous wounds are among the most common soft tissue injuries and are particularly hard to heal in aging. Caloric restriction (CR) is well documented to extend longevity; pharmacologically, profound rejuvenative effects of CR mimetics have been uncovered, especially metformin (MET), resveratrol (RSV), and rapamycin (RAPA). However, locally applied impacts and functional differences of these agents on wound healing remain to be established. Here, we discovered that chronic topical administration of MET and RSV, but not RAPA, accelerated wound healing with improved epidermis, hair follicles, and collagen deposition in young rodents, and MET exerted more profound effects. Furthermore, locally applied MET and RSV improved vascularization of the wound beds, which were attributed to stimulation of adenosine monophosphate‐activated protein kinase (AMPK) pathway, the key mediator of wound healing. Notably, in aged skin, AMPK pathway was inhibited, correlated with impaired vasculature and reduced healing ability. As therapeutic approaches, local treatments of MET and RSV prevented age‐related AMPK suppression and angiogenic inhibition in wound beds. Moreover, in aged rats, rejuvenative effects of topically applied MET and RSV on cell viability of wound beds were confirmed, of which MET showed more prominent anti‐aging effects. We further verified that only MET promoted wound healing and cutaneous integrity in aged skin. These findings clarified differential effects of CR‐based anti‐aging pharmacology in wound healing, identified critical angiogenic and rejuvenative mechanisms through AMPK pathway in both young and aged skin, and unraveled chronic local application of MET as the optimal and promising regenerative agent in treating cutaneous wound defects.

## Introduction

Cutaneous wounds are among the most common soft tissue injuries that require long healing cycle during which severe structural and functional damages or further infection sometimes occur (Shaw & Martin, 2009). Particularly, aging is accompanied by an increasing risk of chronic nonhealing cutaneous wounds, resulting in severe clinical burdens but without effective therapeutics (Sgonc & Gruber, 2013). Currently, the only intervention shown conclusively to counteract aging is caloric restriction (CR) (Fontana & Partridge, 2015), which was also reported to improve wound healing in mammals (Reed *et al*., 1996). Pharmacologically, several CR mimetics have recently been discovered to retard aging and alleviate age‐related pathological changes in various experimental models (Vaiserman *et al*., 2016), particularly metformin (MET) (Barzilai *et al*., 2016), resveratrol (RSV) (Park *et al*., 2012), and rapamycin (RAPA) (Wilkinson *et al*., 2012). Among these anti‐aging agents, surprisingly, RAPA has been documented to inhibit wound healing (Mills *et al*., 2008), probably due to its immunosuppressive capability upon systemic administration (Mills *et al*., 2008; Lamming *et al*., 2013). However, the effects of other CR mimetics MET and RSV on cutaneous wound healing are less understood. Furthermore, considering that local application of agents on skin is more convenient and may exclude potential systemic side effects, elucidating and comparing topical effects of these anti‐aging pharmacological agents on wound healing are of significance to develop clinical relevant strategies for skin defects.

MET, RSV, and RAPA modulate several main signaling pathways mediating CR effects, such as the adenosine monophosphate‐activated protein kinase (AMPK) pathway, the sirtuin 1 (Sirt1) pathway, and the mammalian target of rapamycin (mTOR) pathway (Vaiserman *et al*., 2016). MET, a biguanide agent and AMPK activator, has long been used to treat diabetic hyperglycemia, which substantially impairs wound healing (Madiraju *et al*., 2014). However, controversial results exist regarding whether AMPK activation by MET improves healing of diabetic wounds, in that negative effects on foot ulcers (Ochoa‐Gonzalez *et al*., 2016) and positive effects on gastric ulcers (Baraka & Deif, 2011) upon oral administration of MET have both been reported, suggesting differential effects of MET by systemic and local applications. RSV, a natural polyphenol in red wine and grapes, stimulates both Sirt1 and AMPK pathways, leading to activation of the metabolic regulator peroxisome proliferator‐activated receptor gamma coactivator‐1 alpha (PGC‐1α) (Park *et al*., 2012). As far as we know, putative effects of RSV on cutaneous wound healing have not been substantiated, despite its reported effects on inflammation, fibrogenesis, collagen synthesis, and angiogenesis involved in the wound healing process (Chen & Tseng, 2007; Li *et al*., 2014). RAPA, also called sirolimus, is an mTOR inhibitor and is widely used as an immunosuppressant drug (Lamming *et al*., 2013). During its systemic application, nevertheless, wound healing complications with impaired closure rates often occur (Mills *et al*., 2008), which may be attributed to inhibitory action of mTOR on intraepithelial T lymphocytes in the skin (Mills *et al*., 2008). In a word, functional differences of these CR mimetics on wound healing might exist regarding differential application manners, skin conditions, and pharmacological agents associated with respective molecular targets.

On the other hand, regulation of these longevity‐enhanced pathways may exert general rejuvenative effects on cells not only in aging but also in regeneration of young population (Sharples *et al*., 2015). Nevertheless, their detailed contributions to skin defects and changes with advancing age are still unknown. Therefore, in this study, we aimed to investigate, by local application, the potential impacts and functional differences of MET, RSV, and RAPA on cutaneous wound healing. Meanwhile, we intended to elucidate the optimized approach to regenerate skin defects with putative common mechanism in both young and aged individuals.

## Results

### Effects of locally applied MET, RSV, and RAPA on full‐layer cutaneous wound healing

**Figure 1:**
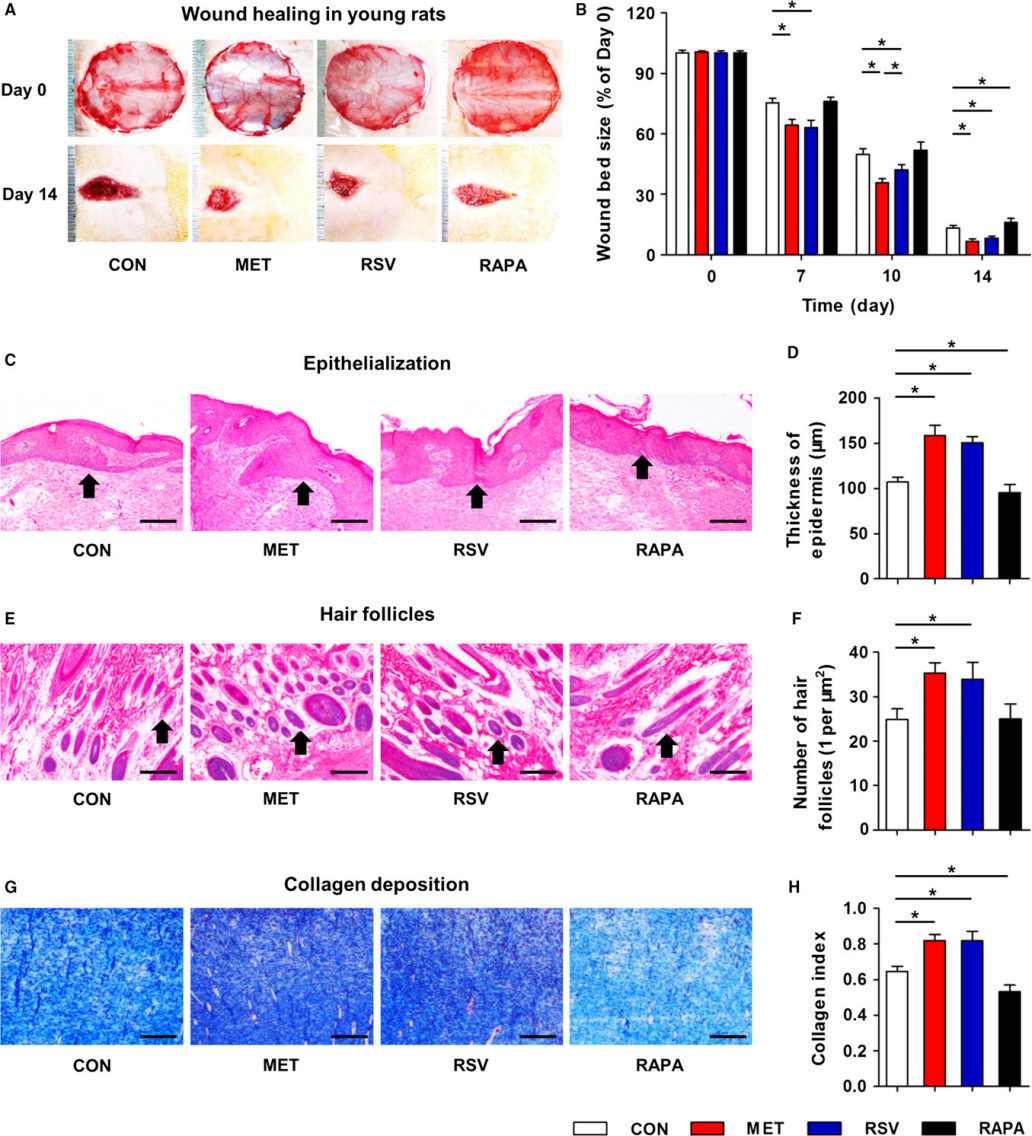
Cutaneous wound healing in young rats with locally applied MET, RSV, and RAPA. (A) Wound bed sizes at indicated time points in 12‐week‐old young rats with locally applied MET, RSV, and RAPA and the dilution control (CON). MET, RSV, and RAPA were respectively diluted at 2, 50 μm and 200 nm in ethanol and applied daily onto the wound beds. One scale of the ruler indicates 1 mm. (B) Quantification of wound bed sizes. (C) HE staining of wound bed samples at Day 14 showing epithelialization with black arrows indicating the epidermis. (D) Quantification of thickness of epidermis. (E) HE staining of wound bed samples at Day 14 showing skin appendages of hair follicles with black arrows indicating the hair follicles. (F) Quantification of number of hair follicles. (G) Masson's trichrome staining of dermal layer at Day 14 showing collagen deposition. (H) Quantification of collagen index. Bars: 100 μm. *n *=* *6 per group. Data represent mean ± SD. *P <* *0.05.

We firstly examined and compared potential effects of MET, RSV, and RAPA on normal skin by local application. As shown, chronic MET and RSV treatments substantially accelerated cutaneous wound healing in young rats, while chronic RAPA treatment slightly inhibited wound healing at Day 14 (Fig. 1A,B). Notably, MET exhibited a little more profound effects compared to RSV as demonstrated by the smaller remaining wound bed sizes at Day 10 (Fig. 1B). For characteristics of the healing wounds, HE staining of the wound bed samples at Day 14 demonstrated thicker epidermis in MET and RSV groups but thinner epidermis in RAPA group, compared to that in control (CON) group (Fig. 1C,D). Further analysis on skin appendages of hair follicles confirmed positive effects of MET and RSV on skin integrity (Fig. 1E,F). Additionally, collagen deposition of different groups showed similar effects, in that MET and RSV substantially promoted while RAPA inhibited collagen deposition (Fig. 1G,H).

Previous studies have reported that these anti‐aging agents, particularly RAPA, may be withdrawn to observe rejuvenation (Blagosklonny, 2008; Leontieva *et al*., 2014). Accordingly, we tested whether intermittent application of MET, RSV, and RAPA exerts promotive effects on wound healing. Interestingly, intermittent administration of MET and RSV did not improve wound healing in young rats, while intermittent administration of RAPA indeed did not inhibit wound healing; however, promotive effects were also not observed after removal of RAPA (Fig. S1, Supporting information). These data suggested that the effects of these anti‐aging agents on wound healing were dependent on persistent usage, which were further confirmed by chronic application in mice, demonstrating positive effects of MET and RSV but negative effects of RAPA (Fig. S2, Supporting information). In addition, prominent effects of MET to promote wound healing were verified in a rabbit model, in which MET, but not RSV and RAPA, significantly accelerated wound closure in the ears (Fig. S3, Supporting information). These findings indicated differential effects of these agents upon chronic application, among which MET showed the greatest power to promote wound healing in normal skin.

### Improved vascularization attributed to stimulation of AMPK pathway in MET‐ and RSV‐ promoted wound healing

**Figure 2:**
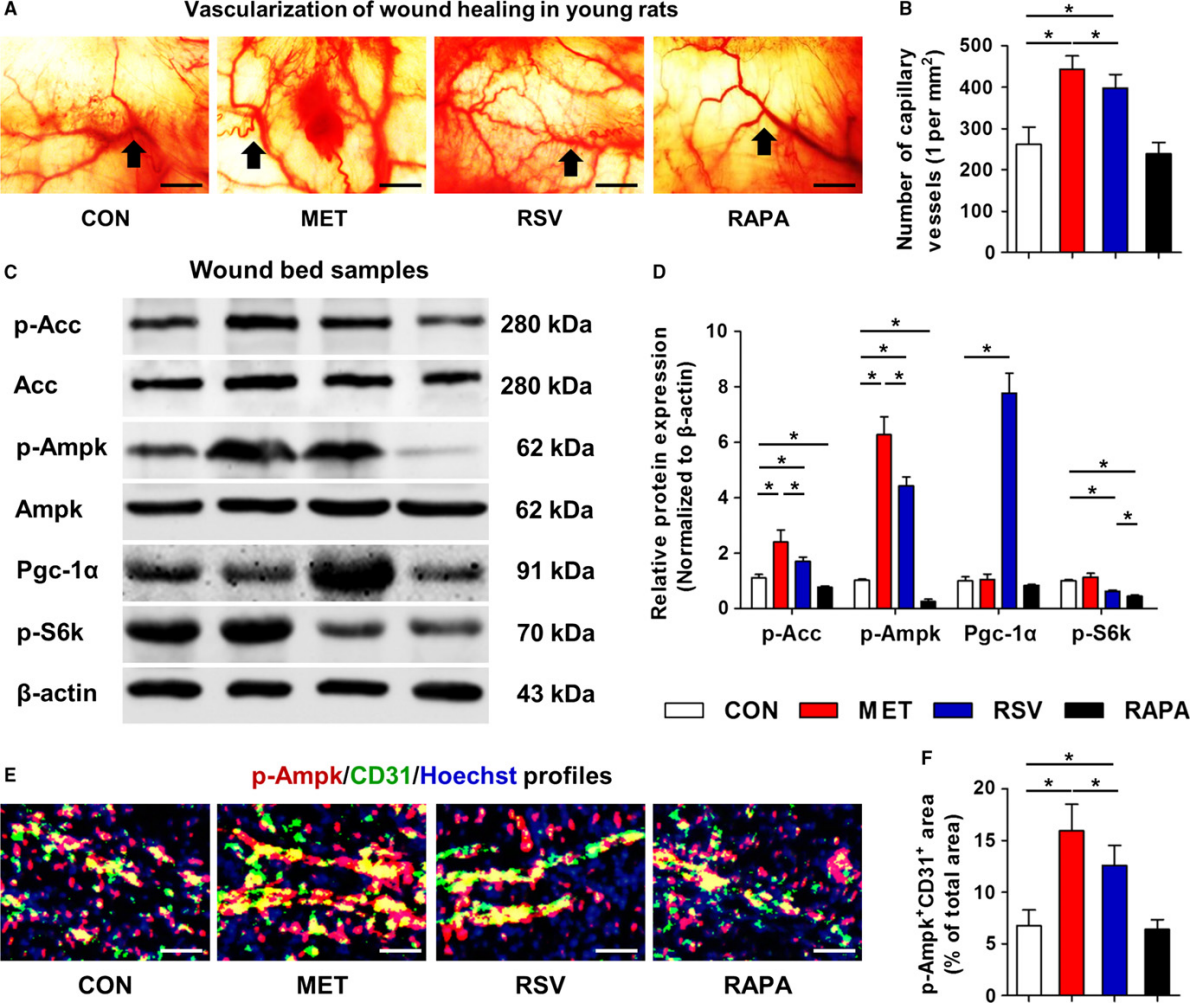
Vascularization and molecular targets in wound bed samples of young rats with locally applied MET, RSV, and RAPA. (A) Vascularization states of cutaneous wound beds at Day 14 with black arrows indicating capillary vessels. (B) Quantification of number of capillary vessels. (C) Western blot analysis in wound bed samples at Day 14 on molecules of AMPK pathway (p‐Acc, Acc, p‐Ampk and Ampk), Sirt1 pathway (Pgc‐1α) and mTOR pathway (p‐S6k). (D) Quantification of western blot data. (E) Immunofluorescent staining of p‐Ampk (Red) and CD31 (Green) in wound bed samples at Day 14. Cell nuclei were counterstained with Hoechst (Blue). (F) Quantification of percentages of p‐Ampk^+^ CD31^+^‐double‐stained area. Bars: 100 μm. *n *=* *6 per group (B, F) and *n *=* *3 per group (D). Data represent mean ± SD. *P <* *0.05.

Vasculature formed in granulation tissues has been recognized as a crucial step of and a critical factor for cutaneous wound healing (Johnson & Wilgus, 2014). Correspondingly, we found that of the vascularization states at Day 14, capillary vessels in chronic MET‐ and RSV‐treated rat wound beds were more than those in CON group, correlated with the improved wound healing. Also, MET exhibited slightly more profound effects to promote angiogenesis compared to RSV (Fig. 2A,B). These vascularization findings were accordingly verified in wound healing of mice, and confirmed by CD31 profiles in granulation tissues, where CD31^+^ cells distributed around microvessels and showed higher percentages in MET and RSV groups (Fig. S4, Supporting information). Effects of chronic RAPA on angiogenesis of wound bed, nevertheless, were not prominent in rats with inhibition in mice (Fig. 2A,B; Fig. S4, Supporting information). These findings suggested overall effects of these agents on the healing wounds were attributed to the modulatory effects on skin angiogenesis.

To explore the molecular mechanisms, we examined the reported key signaling pathways downstream these agents in wound beds, respectively the AMPK, Sirt1 and mTOR pathways (Vaiserman *et al*., 2016) in both rats (Fig. 2C,D) and mice (Fig. S5A,B, Supporting information). Protein expression demonstrated that chronic MET and RSV stimulated AMPK pathway in rodents, as shown by increased p‐Ampk and its downstream component p‐acetyl‐CoA carboxylase (p‐Acc) expression, while chronic RAPA inhibited AMPK pathway in rats. Particularly, highest levels of p‐Acc and p‐Ampk were detected during MET treatments consistently in rats and mice. The expression of Sirt1 pathway mediator, Pgc‐1α, was remarkably upregulated by only RSV in rats, but the changes were not substantial in mice. The phosphorylation of mTOR pathway component ribosomal protein S6 kinase (S6k), however, was inhibited by RAPA in rodents, with differential impacts by MET and RSV in rats and mice (Fig. 2C,D; Fig. S5A,B, Supporting information). Collectively, despite potential contributions of Sirt1 pathway to RSV effects, AMPK pathway may be the key mechanism underlying discrepancy of impacts of these agents on wound healing, while changes of p‐S6k might further explain the inhibitory effects of RAPA.

To further determine the cellular targets in MET‐ and RSV‐ stimulated AMPK pathway, we costained p‐Ampk with the vascular endothelial marker CD31. We discovered that p‐Ampk and CD31 could coexist in wound bed area, labeling a specific shape of vasculature‐like structure (Fig. 2E). Particularly, this colabeled vasculature‐like structure significantly increased upon chronic MET and RSV treatments, while no changes in double‐positively stained area percentages were observed upon chronic RAPA application (Fig. 2F). Furthermore, MET showed more profound effects than RSV, correlated with the promoted vascularization in wound healing. Together, these data indicated improved vascularization attributed to stimulation of AMPK pathway in MET‐ and RSV‐ promoted wound healing.

### Inhibition of AMPK pathway correlated with impaired vascularization in delayed wound healing of aged skin

The above data inspired us to further investigate whether the same mechanism contributes to wound healing deficiency and functions as effective intervention targets of aged skin. Among the above molecular targets, the key role of AMPK pathway in cutaneous wound healing was further uncovered by local application of Compound C, an AMPK signaling inhibitor. We confirmed that Compound C treatment significantly reduced p‐Acc and p‐Ampk protein levels in wound beds (Fig. S5C,D, Supporting information). Moreover, Compound C treatment delayed wound healing in mice, leading to nearly twofold skin defects at Day 14 (Fig. S5E,F, Supporting information). These results suggested that AMPK pathway was the key mediator of wound healing.

**Figure 3:**
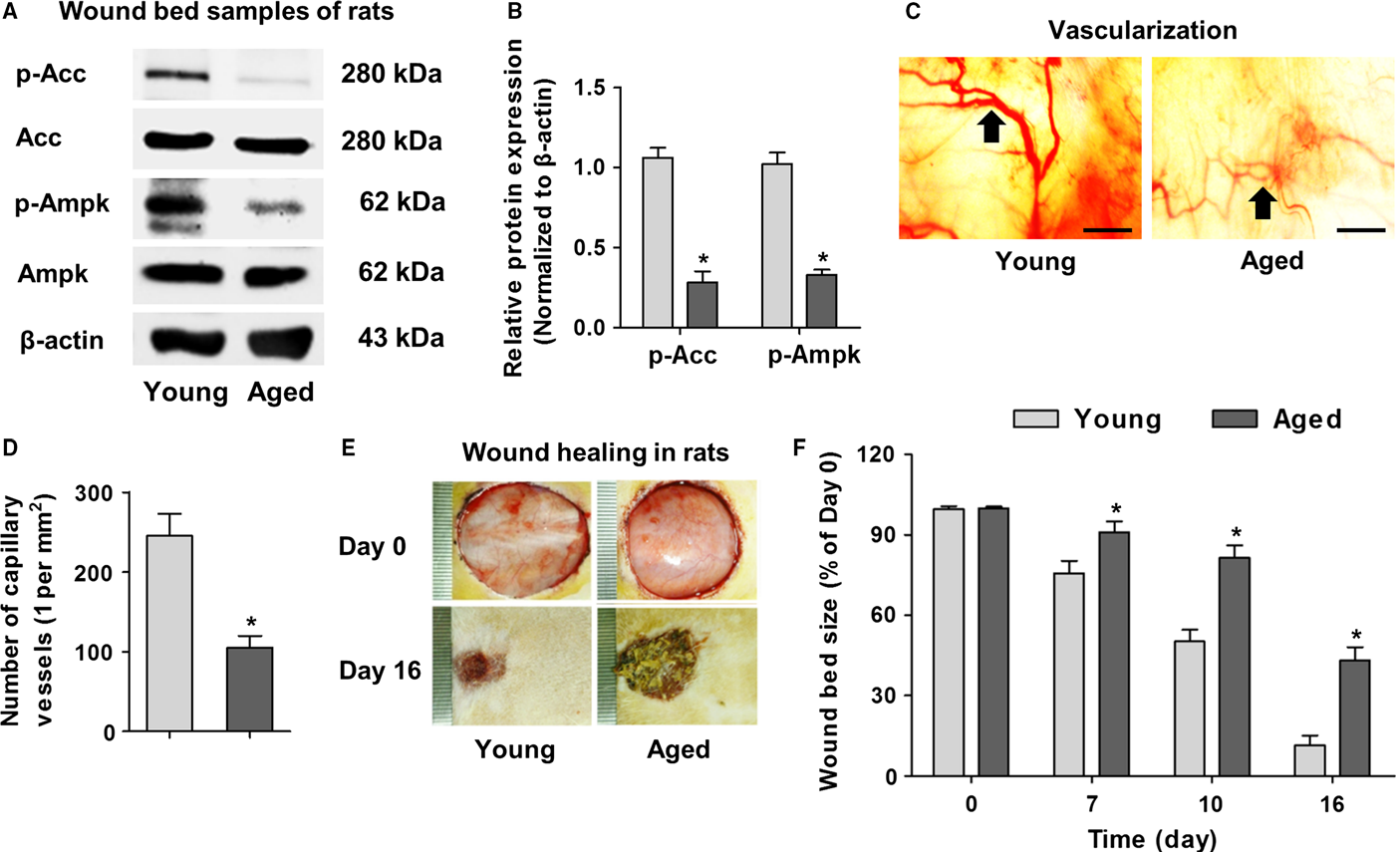
Inhibited AMPK pathway correlated with worse vascularization in delayed wound healing of aged rats. (A) Western blot analysis on molecules of AMPK pathway in wound bed samples at Day 16 in 12‐week‐old (Young) and 18‐month‐old (Aged) rats. (B) Quantification of western blot data. (C) Vascularization states of cutaneous wound beds at Day 16 with black arrows indicating capillary vessels. (D) Quantification of number of capillary vessels. (E) Wound bed sizes at indicated time points in young and aged rats. One scale of the ruler indicates 1 mm. (F) Quantification of wound bed sizes. Bars: 100 μm. *n *=* *3 per group (B) and *n *=* *6 per group (D, F). Data represent mean ± SD. *P <* *0.05.

Then, we discovered in aged rats that the protein expression levels of AMPK pathway components, p‐Acc and p‐Ampk, were significantly downregulated in wound beds compared to those of young rats (Fig. 3A,B). Additionally, we screened and found that aging did not lead to significant changes of Sirt1 and mTOR pathways in skin (Fig. S6A,B, Supporting information). The inhibition of AMPK pathway in aged skin was correlated with impaired vascularization during wound healing, further conforming key roles of AMPK pathway in regulating angiogenesis in wound healing (Fig. 3C,D). We also examined and discovered downregulated p‐Acc and p‐Ampk along epidermis and around hair follicles in wound beds (Fig. S6C,D, Supporting information). As a result, cutaneous wound healing in aged rats was delayed (Fig. 3E,F), with thinner epidermis, less hair follicles and deficient collagen deposition (Fig. S6E–J, Supporting information) compared to their young counterparts. These results further provided AMPK pathway as the potential targets to promote wound healing in aged skin.

### Local MET and RSV treatments prevent age‐related AMPK suppression and angiogenic inhibition in wound beds

**Figure 4:**
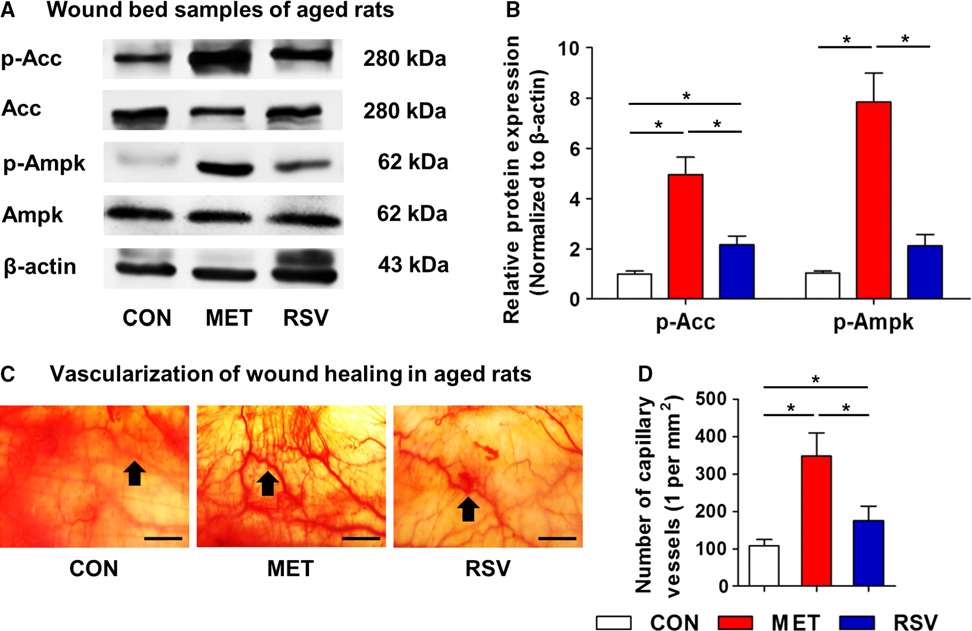
Stimulation of AMPK pathway and pro‐vascularization effects of MET and RSV during wound healing in aged skin. (A) Western blot analysis on molecules of AMPK pathway in wound bed samples at Day 14 in aged rats with locally applied MET and RSV and the dilution control (CON). (B) Quantification of western blot data. (C) Vascularization states of cutaneous wound beds at Day 14 with black arrows indicating capillary vessels. (D) Quantification of number of capillary vessels. Bars: 100 μm. *n *=* *3 per group (B) and *n *=* *6 per group (D). Data represent mean ± SD. *P <* *0.05.

Considering MET and RSV stimulated AMPK signaling in young rodents, we further applied these agents, but not RAPA, in aged skin. Western blot analysis demonstrated upregulation of both p‐Acc and p‐Ampk by chronic MET and RSV in wound beds of aged rats (Fig. 4A). Notably, statistical analysis showed more powerful effects of MET compared to RSV (Fig. 4B). As expected, vascularization states were also promoted by chronic MET and RSV treatments (Fig. 4C). Particularly, MET application led to a more than threefold increase in the vasculature in wound beds of aged rats. These findings indicated remarkable potential of MET, as well as RSV, in improving wound healing of aged skin.

### Local MET and RSV treatments alleviate aging and rejuvenate cutaneous cell viability in aged skin

Next, we investigated whether chronic MET and RSV could indeed exhibit anti‐aging effects in cutaneous wound healing. We confirmed that MET and RSV promoted the cellularity of granulation tissues, as depicted by the proliferative marker proliferating cell nuclear antigen (PCNA) staining in mice. On the contrary, chronic RAPA treatment inhibited proliferative cells along epidermis and around hair follicles (Fig. S7A,B, Supporting information). Furthermore, for mRNA expression in wound beds, MET upregulated *cyclin D1* (*Ccnd1*), a proliferative promoter, and downregulated cell cycle inhibitors *P53*,* P21*, and *P16*, which are also known as cell senescent markers (Sui *et al*., 2016a). RSV also promoted *Ccnd1* and inhibited *P53*,* P21*, and *P16*, but its effects were not as powerful as those of MET. The effects of RAPA, however, were paradoxical in suppressive effects on *P53* and *P21* but stimulatory effects on *P16*, which might result in the paralleled *Ccnd1* level (Fig. S7C, Supporting information).

**Figure 5:**
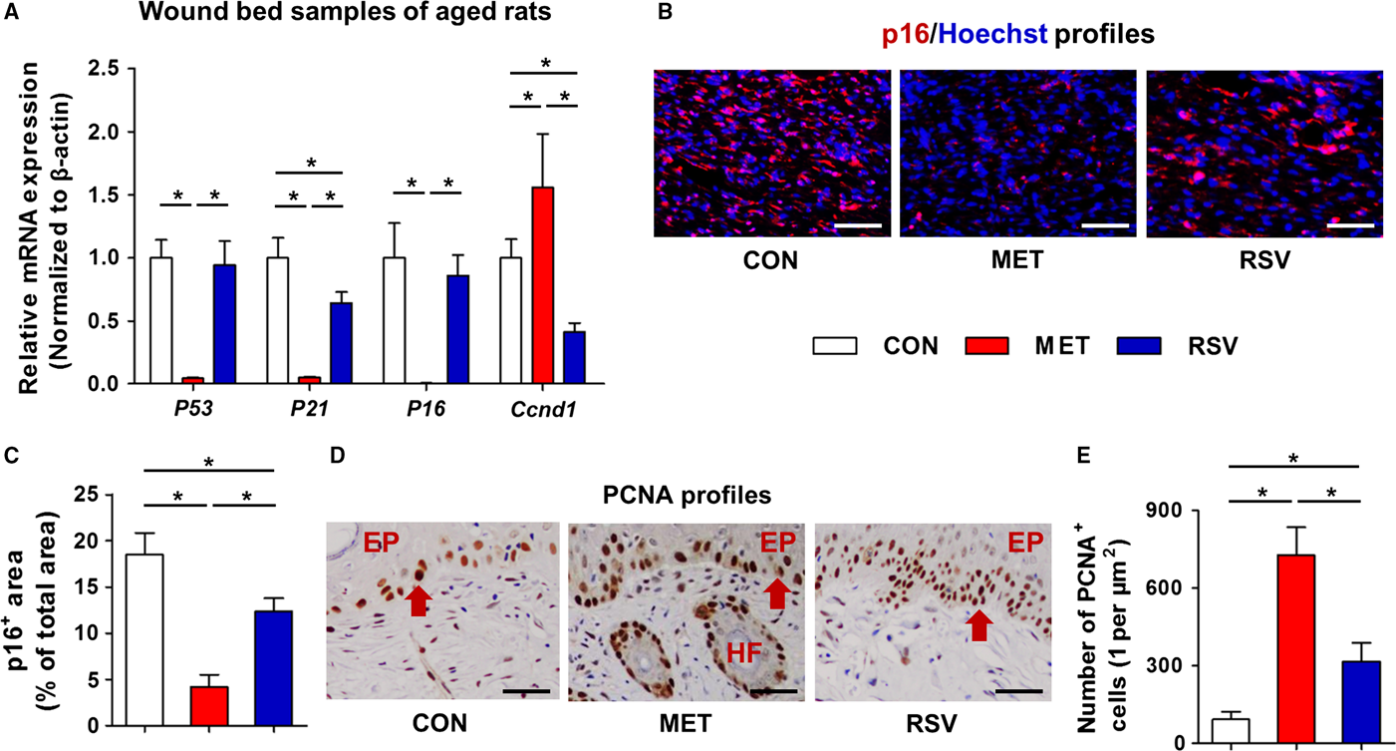
Anti‐aging effects of MET and RSV during wound healing in aged skin. (A) qRT–PCR analysis on mRNA expression levels of the proliferative marker *Ccnd1* and senescent markers *P53*,* P21*, and *P16* in wound bed samples at Day 14 in aged rats with locally applied MET and RSV and the dilution control (CON). (B) Immunofluorescent staining of p16 expression (Red) in wound bed samples at Day 14 in aged rats (counterstained by Hoechst, Blue). (C) Quantification of percentages of p16^+^ area. (D) Immunohistochemistry staining of PCNA expression, with red arrows indicating the positively stained cells along the epidermis (EP) and around hair follicles (HF). (E) Quantification of number of PCNA ^+^ cells. Bars: 100 μm. *n *=* *3 per group (A) and *n *=* *6 per group (C, E). Data represent mean ± SD. *P <* *0.05.

In aged skin of rats, moreover, analysis on proliferative and senescent markers revealed significant rejuvenative effects of MET, while RSV showed paradoxical effects in suppression of both *P21* and *Ccnd1* (Fig. 5A). Anti‐aging effects of these agents were also verified in wound beds with suppression of p16^+^ area, and MET exhibited more profound anti‐aging effects (Fig. 5B,C). PCNA staining in wound samples further confirmed these results, demonstrating increases in proliferative cells along epidermis and around hair follicles by MET and RSV treatments, of which MET showed more prominent effects (Fig. 5D,E). These findings indicated that local MET and RSV treatments indeed ameliorated aging in cutaneous wound healing, of which MET may exhibit more profound anti‐aging effects.

### Local application of MET strongly promotes wound healing in aged skin

**Figure 6:**
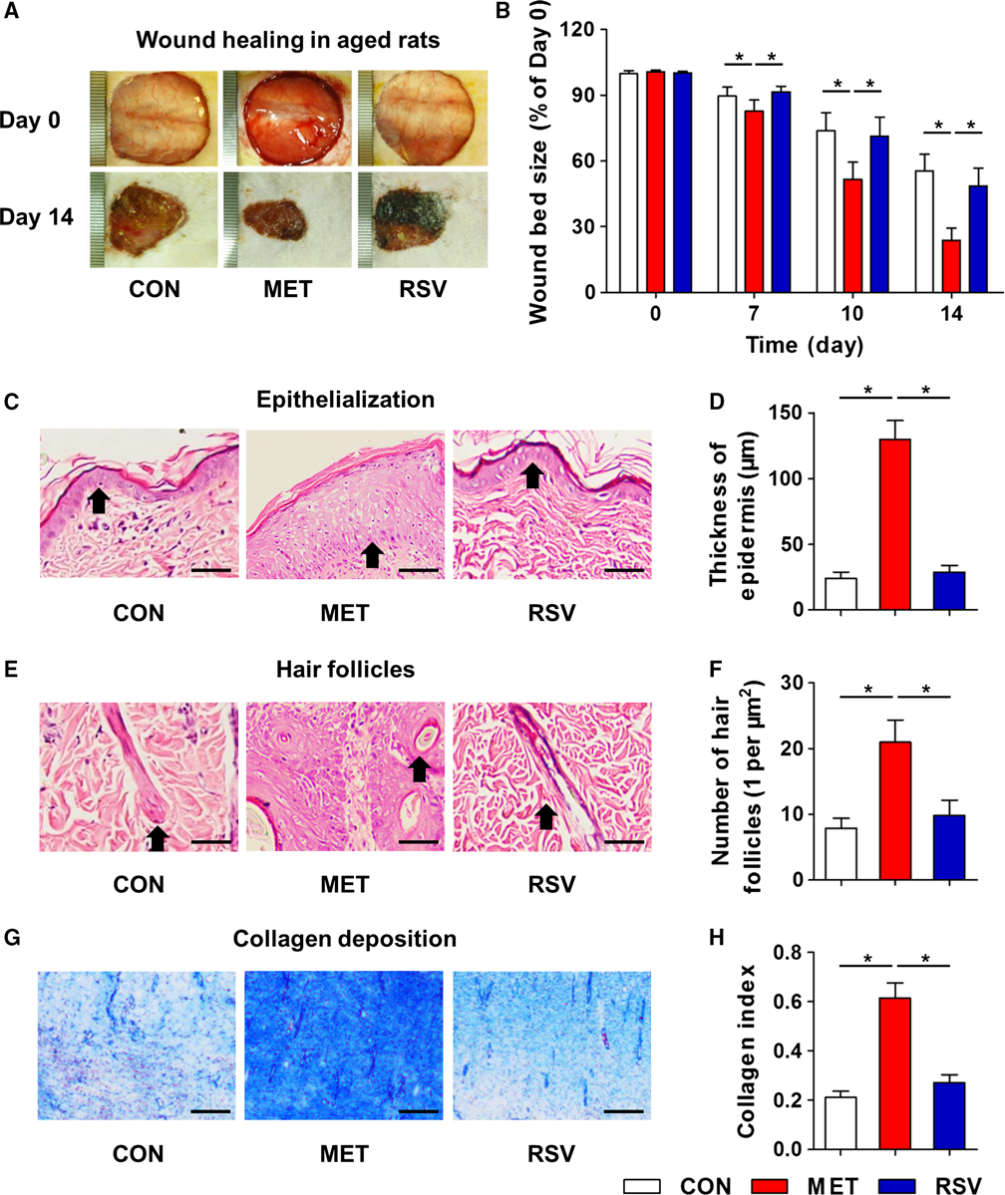
Local application of MET promoted full‐layer cutaneous wound healing in aged rats. (A) Wound bed sizes at indicated time points in 18‐month‐old aged rats with locally applied MET and RSV and the dilution control (CON). MET and RSV were respectively diluted at 2 and 50 μm in ethanol and applied daily onto the wound beds. One scale of the ruler indicates 1 mm. (B) Quantification of wound bed sizes. (C) HE staining of wound bed samples at Day 14 showing epithelialization with black arrows indicating the epidermis. (D) Quantification of thickness of epidermis. (E) HE staining of wound bed samples at Day 14 showing skin appendages of hair follicles with black arrows indicating the hair follicles. (F) Quantification of number of hair follicles. (G) Masson's trichrome staining of dermal layer at Day 14 showing collagen deposition. (H) Quantification of collagen index. Bars: 100 μm. *n *=* *6 per group. Data represent mean ± SD. *P <* *0.05.

These findings promoted us to investigate effects of locally applied MET and RSV on wound healing in aged skin. With regard to cutaneous wound healing rates, MET significantly accelerated wound healing in aged rats, while no obvious effects were detected after RSV application (Fig. 6A,B). HE staining further demonstrated a substantial increase in the thickness of epidermis by MET treatment in aged skin, with almost fourfold thicker compared to that of CON and RSV groups (Fig. 6C,D). Skin appendage analysis on hair follicles confirmed that only MET promoted cutaneous integrity in aged skin (Fig. 6E,F). Also, only MET facilitated collagen deposition of aged akin (Fig. 6G,H). Together, these results clarified effects of anti‐aging pharmacology in aged rats, indicating that local chronic application of MET strongly promotes wound healing.

## Discussion

CR rescues impaired wound healing (Reed *et al*., 1996), but locally applied impacts and potential functional differences of CR mimetics MET, RSV, and RAPA (Park *et al*., 2012; Wilkinson *et al*., 2012; Vaiserman *et al*., 2016) on wound healing remain to be established. Here, we investigated and compared effects of these anti‐aging pharmacological agents on cutaneous wound healing by local application. We discovered that MET and RSV, but not RAPA, improved wound healing in young rodents. Particularly, MET exhibited more profound effects, and further exerted prominent regenerative effects in aged skin. We have also identified critical angiogenic and rejuvenative mechanisms through AMPK pathway in wound healing of both young and aged skin. These findings unraveled local application of MET as the promising therapeutic agent in wound defects.

The skin is the biggest organ of the human being and the healing of a cutaneous wound displays an extraordinary feature of regeneration in nature (Reinke & Sorg, 2012). Nevertheless, aging induces structural and functional alterations in skin, including a decrease in dermal thickness, decline in collagen contents and a loss of elasticity (Sgonc & Gruber, 2013), which underlie impaired wound healing in aged individuals. Notably, in both young and aged populations, current pharmacological therapies based on glucocorticoids yield controversial results (Hofman *et al*., 2007). The ability of CR to alleviate age‐related pathologies has been documented in various mammal organs (Fontana & Partridge, 2015) including the skin, in which caloric‐restricted mice showed enhanced epithelialization and collagen synthesis in wound repair (Reed *et al*., 1996). Therefore, it is reasonable to assume that anti‐aging by CR mimetics could promote wound healing and similar rejuvenation could be exerted in both young and aged skin though the same mechanism. In this study, we clarified that differential effects exist among CR mimetics MET, RSV, and RAPA upon local application, in that MET exhibited favorable effects but RAPA exerted potential detrimental effects, while RSV could be conditionally beneficial. It is interesting that the potential inhibitory effects of chronic RAPA on wound healing were detected slightly and only as late events in wound healing of both rodents and rabbits, while particularly MET tend to show beneficial effects at an early stage. This functional discrepancy might be attributed to differential effects of these agents on different wound healing phases, as discussed later. Also intriguing is that anti‐aging does not guarantee regeneration. Although aging is recognized by impaired regenerative capacity, several recent studies have revealed that regeneration might not be affected by aging (Eguchi *et al*., 2011), and cell senescence during development may even contribute to tissue remodeling in vertebrates (Storer *et al*., 2013). Together with these findings, our results suggested a revisit on the correlations between aging and regeneration.

Wound healing is highly orchestrated that could be divided into three major overlapping phases: inflammation, proliferation and remodeling (Reinke & Sorg, 2012). During these steps, various types of cells including immune cells, epithelial cells, endothelial cells and fibroblasts migrate or proliferate and coordinate in driving key processes for skin regeneration, such as reepithelialization, neovascularization, and extracellular matrix production on the basis of granulation tissues (Reinke & Sorg, 2012). RAPA, an immunosuppressant (Lamming *et al*., 2013), has been shown to impair skin‐resident T cells and reduce T‐cell secretion of growth factors that are important for the subsequent wound healing at later phases (Mills *et al*., 2008). In addition, although reports by Blagosklonny *et al*. documented lifespan extension by RAPA upon intermittent application to alleviate the side effects (Leontieva *et al*., 2014), neither chronic nor intermittent administration of RAPA in our study exerted beneficial effects on wound healing. Nevertheless, a further open question that remains is whether administration of RAPA prior to wounding would be protective. The effects of RSV, surprisingly, range widely from inflammation to fibrogenesis, angiogenesis, and collagen synthesis (Chen & Tseng, 2007; Li *et al*., 2014). However, contradictory results exist as to whether RSV exerts pro‐ or anti‐ effects on angiogenesis and cell viability (Chen & Tseng, 2007), which might be dose‐dependent (Girbovan *et al*., 2016). Here, we demonstrated that effects of RSV on wound healing are indeed conditionally beneficial. At different concentrations or observed after longer period may prove therapeutic effects of RSV on aged wounds.

The most important finding of this study is to uncover local application of MET as the promising therapeutics in wound defects of both young and aged skin. Mechanistically, MET has been reported to substantially affect angiogenesis to regulate cell proliferation (Dallaglio *et al*., 2014). Nevertheless, pro‐angiogenic effects of MET have also been reported, particularly in tissue regeneration processes upon persistent administration (Liu *et al*., 2014b). Here, pro‐angiogenesis by locally applied MET was further confirmed post‐wound excision. Molecularly, we discovered that angiogenic effects of MET were mediated by activation AMPK pathway. AMPK signaling, which senses decreases in energy charge (Zhou *et al*., 2001), is known to stimulate vascular endothelial growth factor (VEGF) expression and is necessary to angiogenesis (Ouchi *et al*., 2005). As further shown in this study, AMPK stimulation not only by MET but also by RSV primarily occurred in CD31^+^ endothelial cells to promote vascularization of the healing wounds, despite potential cellular targets of epithelial cells (Sun *et al*., 2017) and immune cells (Yang & Chi, 2015). The key roles of AMPK pathway in angiogenesis and wound healing were also uncovered with advancing age and by Compound C‐based inhibition in rodents. Furthermore, rejuvenative effects of MET on aged skin were substantial, which may be secondary to angiogenic effects or direct contribution of AMPK pathway that is significant to mediate systemic anti‐aging impacts of MET (Barzilai *et al*., 2016).

Several intriguing issues arise with our present findings that are interesting to be addressed in future studies. As stated, it should be cautious that systemic administration of these agents may result in more unpredictable side effects and failed therapeutics in the skin, potentially underlying differential effects of MET by local and systemic applications (Baraka & Deif, 2011; Ochoa‐Gonzalez *et al*., 2016). Therefore, topically targeting AMPK by MET may become a feasible approach to the chronic nonhealing wounds in both young and aged population, or function as a useful supplement to current treatments such as glucocorticoids (Hofman *et al*., 2007) and epidermal growth factor (EGF) (Brown *et al*., 1989). In this regards, chronic rather than intermittent administration should be applied, but whether the effects of MET are additive to or blinded by these treatments in combination usage remain to be explored. Furthermore, considering the correlated changes of AMPK downstream targets such as phosphorylation of Acc (Zhou *et al*., 2001), direct manipulation of these functional targets may also bring beneficial effects on cutaneous wound healing in future works. Besides, RSV could also activate AMPK signaling, but its effects are not as powerful as MET according to our data, particularly in aged skin. Another mediator of RSV effects, the Sirt1 pathway component Pgc‐1α (Park *et al*., 2012), may further explain the effects of RSV on wound healing, but at least the less effective stimulation on AMPK pathway limits RSV application on aged wounds. Additionally, mTOR pathway was revealed to be particularly suppressed by chronic administration of its inhibitor, RAPA (Lamming *et al*., 2013). Other than key function of mTOR in skin‐resident T cells (Mills *et al*., 2008), mTOR pathway is also important to promote epithelial cell proliferation (Cai *et al*., 2012) and angiogenesis (Yu *et al*., 2014), thus explaining potential modulatory effects of MET and RSV on mTOR activity that may be secondary to activation of cell viability by these agents. Accordingly, stimulation of mTOR may provide beneficial effects on wound healing similar to AMPK activation, but the specific mTOR stimulation agents need to be established.

In summary, differential effects exist among CR‐based anti‐aging pharmacological agents of MET, RSV, and RAPA in cutaneous wound healing. Our findings further identify critical angiogenic and rejuvenative mechanisms through AMPK pathway in both young and aged skin, and unravel chronic local application of MET as the optimal and promising regenerative agent in treating cutaneous wound defects.

## Experimental procedures

### Animals

All experiments were approved by Fourth Military Medical University and were performed following the Guidelines of Intramural Animal Use and Care Committee of Fourth Military Medical University, the ARRIVE guidelines and the NIH Guide for the Care and Use of Laboratory Animals.

Twelve‐week‐old female C57BL/6 mice, 12‐week‐old and 18‐month‐old female Sprague‐Dawley rats, and 6‐month‐old female New Zealand White rabbits (Laboratory Animal Center, Fourth Military Medical University, Xi'an, China) were used. Animals were randomly assigned to different experimental groups, maintained with good ventilation and a 12‐h light/dark cycle, and were kept feeding and drinking *ad libitum* before being sacrificed.

### Acute full‐layer cutaneous wound modeling

Full thickness excision wound creation was performed based on our previous report (Liu *et al*., 2014a). Experimental animals were anesthetized by intraperitoneal injection of 1% pentobarbital sodium. The dorsal skin of mice and rats and ear skin of rabbits were shaved and scrubbed, and full‐layer cutaneous wounds were carefully made using ophthalmic scissors under sterile surgical conditions. The position of wounds of mice and rats was located on the top half of the back across the midline of dorsum surface. The wounds of rabbits were created down to the bare cartilage on the ventral side of ears. Original wound bed sizes for mice, rats and rabbits were respectively 2, 3 and 2 cm in diameter.

### Agents

MET (Sigma‐Aldrich, St. Louis, MO, USA), RSV (Sigma‐Aldrich), RAPA (Tokyo Chemical Industry, Tokyo, Japan) and Compound C (Santa Cruz Biotechnology, Santa Cruz, CA, USA) were used in this study. Ethanol was selected as the dilution based on previous reports (Xing *et al*., 2015). The working concentrations of these agents were determined based on previous studies using these agents for skin, which were further confirmed in our preliminary tests using a small population of mice to verify that the concentrations were set at the effective levels for wound treatment: MET at a concentration of 2 μm (Wu *et al*., 2013), RSV at 50 μm (George *et al*., 2011), RAPA at 200 nm (Checkley *et al*., 2011), and Compound C at 10 μm (Cao *et al*., 2008). Agents were locally applied using pipettes onto the wound beds at 100 μL per time in mice and rabbits and at 225 μL per time in rats. The amounts of agents used were set according to the wound bed areas. CON group accepted 100‐ or 225‐μL ethanol. For chronic application, agents were administered 1 time every day. For intermittent application, agents were administered every other day three times during 1 week followed by a treatment‐free week, according to previous methods (Leontieva *et al*., 2014).

### Wound healing assessment

Wound bed sizes during approximately 2‐week experimental periods were observed daily and imaged at indicated time points by digital camera. Wound bed sizes were then quantified by imagej software (National Institute of Health, Bethesda, MD, USA), and data were calculated as follows: (actual wound area/original wound area) ×100%.

### Vascularization assessment

After sacrifice of mice and rats at indicated time points, biopsies of the original wound area with the healed and the remaining wound beds were sampled and placed in culture dishes with inner face down. Vascularization states of each specimen were evaluated under incandescent illumination and photographed by digital camera. Number of capillary vessels in the healed wound bed area was quantified by imagej software, as stated (An *et al*., 2015).

### Histological analysis

Wound bed biopsies were evenly divided into four parts, respectively, for paraffin‐embedded and frozen sections, and mRNA and protein extractions. For histological analysis, specimens were fixed overnight with 4% paraformaldehyde, embedded in paraffin, sectioned to 5‐μm‐thick sections, and then deparaffinized. HE staining was conducted as previously described (An *et al*., 2015), and the thickness of epidermis and the number of hair follicles in the healed wound beds were analyzed using ImageJ software. For analysis of collagen deposition, Masson's trichrome staining was performed with a commercial kit (Sigma‐Aldrich) according to manufacturer's instructions. Collagen index within the healed wound beds was evaluated as reported (Olbrich *et al*., 2005).

### Immunohistochemistry

Immunohistochemistry was performed according to our published methods (Chen *et al*., 2016). Deparaffinized sections were treated with 0.25% trypsin (MP Biomedicals, Santa Ana, CA, USA) for 30 min at 37 °C for antigen retrieval, washed, and treated with 3% hydrogen peroxide for 20 min at 37 °C. Sections were blocked with 5% bovine serum albumin (BSA) (Sigma‐Aldrich) in PBS for 2 h at room temperature. Sections were then stained with primary antibodies overnight at 4 °C as follows: a rabbit anti‐mouse CD31 antibody at a concentration of 1:100 (Abcam, Cambridge, UK); a rabbit anti‐mouse/rat PCNA antibody at a concentration of 1:100 (Abcam); a rabbit anti‐rat p‐Ampk antibody at a concentration of 1:200 (Cell Signaling Technology, Danvers, MA, USA); and a rabbit anti‐rat p‐Acc antibody at a concentration of 1:50 (Cell Signaling Technology). The sections were then stained by a goat anti‐rabbit secondary antibody (Cell Signaling Technology) for 30 min at room temperature at a concentration of 1:200. Subsequently, an HRP‐based Dako REAL™ EnVision™ Detection System (Agilent Technologies, Santa Clara, CA, USA) was used to detect the immunoactivity, followed by counterstaining with hematoxylin (Sigma‐Aldrich). Quantification of the number of positively stained cells or percentages of positively stained area over total area was performed using the imagej (National Institute of Health, Bethesda, MD, USA) software.

### Immunofluorescent staining

Immunofluorescent staining was performed according to our previous work (Sui *et al*., 2016b). Specimens were fixed overnight with 4% paraformaldehyde, cryoprotected with 30% sucrose, embedded in the optimal cutting temperature compound, snap‐frozen, and sectioned into 15‐μm sagittal sections (CM1950, Leica, Solms, Germany). Sections were blocked with 5% BSA (Sigma‐Aldrich) dissolved in PBS for 1 h at room temperature. Sections were stained with primary antibodies for 2 h at room temperature as follows: a rabbit anti‐rat p16 antibody (Abbiotec, San Diego, CA, USA) at a concentration of 1:200; and a goat anti‐rat CD31 antibody (Abcam) costained with a rabbit anti‐rat p‐Ampk antibody (Cell Signaling Technology) at both concentrations of 1:200. The sections were then stained by a goat anti‐rabbit‐PE secondary antibody and/or a donkey anti‐goat‐FITC secondary antibody for 30 min at room temperature at concentrations of 1:200. Sections were counterstained with Hoechst (Sigma‐Aldrich) for 3 min at room temperature. Quantification of the number of positively stained cells or percentages of positively stained area over total area was performed using the ImageJ software.

### Quantitative real‐time polymerase chain reaction (qRT–PCR) analysis

Total RNA was collected from mice and rat wound bed samples. RNA was extracted by the addition of TRIzol Reagent (Takara, Tokyo, Japan), grinded under liquid nitrogen, and purified by phenol–chloroform extraction. cDNA synthesis and PCR procedures were performed as described (Chen *et al*., 2016). The primer sequences are listed in Table S1 (Supporting Information).

### Western blot

Western blot was performed for mice and rat wound bed samples as previously described (Sui *et al*., 2017). Tissue lysates were prepared using the Cell Lysis Buffer (Beyotime, Shanghai, China). Proteins were extracted, loaded on sodium dodecyl sulfate–polyacrylamide gels, transferred to polyvinylidene fluoride membranes (Millipore, MA, USA), and blocked with 5% BSA (Sigma‐Aldrich) in PBST (PBS with 0.1% Tween) for 2 h at room temperature. The membranes were incubated overnight at 4°C with the following primary rabbit anti‐mouse/rat antibodies: for p‐Acc, Acc, p‐Ampk, Ampk and p‐S6k (all from Cell Signaling Technology) at all concentrations of 1:1000; for Pgc‐1α (Abcam) at a concentration of 1:1000; and for β‐actin (Abcam) at a concentration of 1:4000. The membranes were then incubated with peroxidase‐conjugated goat anti‐rabbit secondary antibodies (Boster, Shenyang, China) at a concentration of 1:40 000*g* for 1 h at room temperature. The blotted bands were visualized using an enhanced chemiluminescence kit (Amersham Biosciences, Piscataway, NJ, USA) and a gel imaging system (5500; Tanon, Shanghai, China). The gray values of the bands were analyzed using the imagej software.

### Statistical analysis

Results are represented as the mean ± standard deviation (SD). Data were analyzed using two‐tailed Student's *t*‐tests (for two‐group comparisons) or one‐way analysis of variance (ANOVA) followed by Newman–Keuls post hoc tests (for multiple‐group comparisons) in the graphpad prism 5.01 software (GraphPad Software Inc., La Jolla, CA, USA). Values of *P *<* *0.05 were considered statistically significant.

## Funding

This work was supported by The National Key Research and Development Program of China (2016YFC1102900 and 2016YFC1101400) and The General Program of National Natural Science Foundation of China (81570937 and 81470710).

## Author contributions

P.Z., B.D.S., and N.L. contributed equally to the study design, experimental work, data analysis, data interpretation, and manuscript preparation. Y.J.L. and C.X.Z. contributed to the experimental work and revised the manuscript. Y.B.L. and W.T.H. contributed to the data interpretation. C.H.Z., J.C., D.L.P., D.D.F., and K.X. contributed to the data interpretation. C.H.H. and Y.J. conceived and supervised the study. All authors have reviewed and approved the final version of the manuscript.

## Conflict of interest

The authors state no conflict of interest.

## Supporting information


**Fig. S1** Cutaneous wound healing in rats with intermittent application of MET, RSV, and RAPA.
**Fig. S2** Cutaneous wound healing in mice with locally applied MET, RSV, and RAPA.
**Fig. S3** Full‐layer cutaneous wound healing in rabbits with locally applied MET, RSV, and RAPA.
**Fig. S4** Vascularization of the healing wounds in mice with locally applied MET, RSV, and RAPA.
**Fig. S5** AMPK pathway plays key roles in promoting wound healing.
**Fig. S6** Impaired wound healing ability with inhibited AMPK signaling pathway in aged skin.
**Fig. S7** Anti‐aging effects of locally applied MET, RSV, and RAPA during wound healing in mice.
**Table S1** Primer sequences in the present study for mice and rats.
