# Corrigendum to: Anti‐aging pharmacology in cutaneous wound healing: Effects of metformin, resveratrol, and rapamycin by local application

**DOI:** 10.1111/acel.13561

**Published:** 2022-02-02

Pan Zhao, Bing‐Dong Sui, Nu Liu, Ya‐Jie Lv, Chen‐Xi Zheng, Yong‐Bo Lu, Wen‐Tao Huang, Cui‐Hong Zhou, Ji Chen, Dan‐Lin Pang, Dong‐Dong Fei, Kun Xuan, Cheng‐Hu Hu, Yan Jin, *Aging cell*, *16*(5), 1083–1093. https://doi.org/10.1111/acel.12635

In Zhao et al. (2017), the authors noticed the following errors:

1. There was an error in displaying Acc bands in Figure 2c.
2. The descriptions about the experimental groups in Figure 2c were missing.

The corrected Figure 2, together with the accompanying legends, was shown below.

These corrections do not affect the conclusions of the above paper. The authors would like to apologize for the inconvenience caused.

**FIGURE 2:**
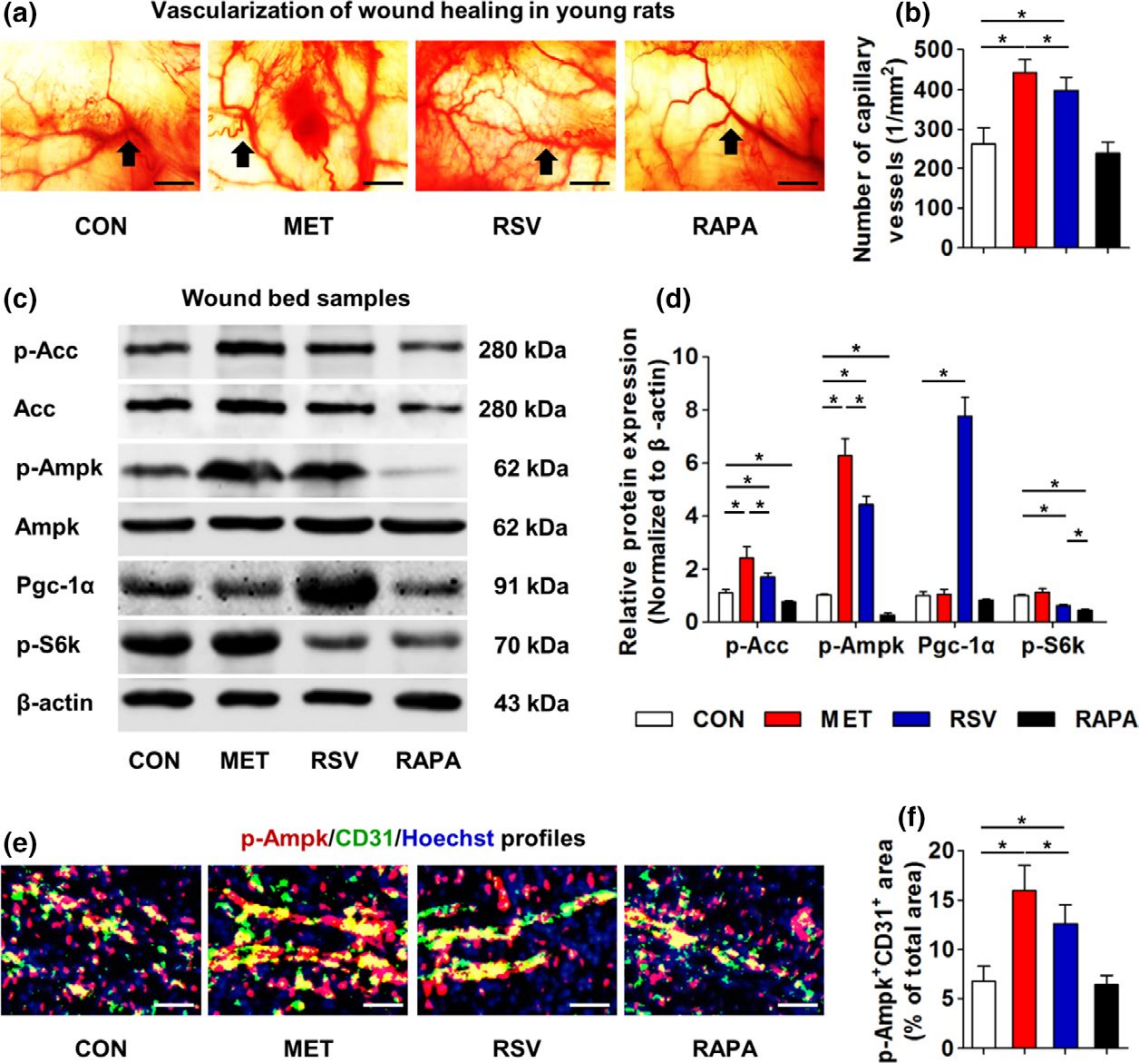
Vascularization and molecular targets in wound bed samples of young rats with locally applied MET, RSV, and RAPA. (a) Vascularization states of cutaneous wound beds at Day 14 with black arrows indicating capillary vessels. (b) Quantification of number of capillary vessels. (c) Western blot analysis in wound bed samples at Day 14 on molecules of AMPK pathway (p‐Acc, Acc, p‐Ampk, and Ampk), Sirt1 pathway (Pgc‐1α), and mTOR pathway (p‐S6k). (d) Quantification of Western blot data. (e) Immunofluorescent staining of p‐Ampk (Red) and CD31 (Green) in wound bed samples at Day 14. Cell nuclei were counterstained with Hoechst (Blue). (f) Quantification of percentages of p‐Ampk^+^CD31^+^ double stained area. Bars: 100 μm. *n* = 6 per group (b, f) and *n* = 3 per group (d). Data represent mean ± SD. *p < 0.05
