# Supplementary material for: Anti‐aging pharmacology in cutaneous wound healing: effects of metformin, resveratrol, and rapamycin by local application
Source: Aging Cell. 2017 Jul 5;16(5):1083–93. doi: 10.1111/acel.12635 (PMC5595695; doi:10.1111/acel.12635)
Supplement: Supplementary file 1 — Fig. S1 Cutaneous wound healing in rats with intermittent application of MET, RSV, and RAPA. Fig. S2 Cutaneous wound healing in mice with locally applied MET, RSV, and RAPA. Fig. S3 Full‐layer cutaneous wound healing in rabbits with locally applied MET, RSV, and RAPA. Fig. S4 Vascularization of the healing wounds in mice with locally applied MET, RSV, and RAPA. Fig. S5 AMPK pathway plays key roles in promoting wound healing. Fig. S6 Impaired wound healing ability with inhibited AMPK signaling pathway in aged skin. Fig. S7 Anti‐aging effects of locally applied MET, RSV, and RAPA during wound healing in mice. Table S1 Primer sequences in the present study for mice and rats. [file ACEL-16-1083-s001.docx]

***SUPPORTING INFORMATION***

**Anti-aging Pharmacology in Cutaneous Wound Healing: Effects of Metformin, Resveratrol and Rapamycin by Local Application**

Pan Zhao,^1, 2†^ Bing-Dong Sui,^1, 3†^ Nu Liu,^1, 3, 4†^ Ya-Jie Lv,^1, 5^ Chen-Xi Zheng,^1, 3^ Yong-Bo Lu,^2^ Wen-Tao Huang,^2^ Cui-Hong Zhou,^1, 2^ Ji Chen,^1^ Dan-Lin Pang,^1, 3^ Dong-Dong Fei,^1^ Kun Xuan,^1, 3^ Cheng-Hu Hu^1, 2*^ and Yan Jin^1, 3*^

^1^ State Key Laboratory of Military Stomatology, Center for Tissue Engineering, Fourth Military Medical University, Xi’an, Shaanxi, 710032, China.

^2^ Xi’an Institute of Tissue Engineering and Regenerative Medicine, Xi’an, Shaanxi, 710032, China.

^3^ Research and Development Center for Tissue Engineering, Fourth Military Medical University, Xi’an, Shaanxi 710032, China.

^4^ Department of Periodontology, Stomatological Hospital, Zunyi Medical College, Zunyi, Guizhou 563003, China.

^5^ Department of Dermatology, Tangdu Hospital, Fourth Military Medical University, Xi’an, Shannxi 710069, China.

^†^ These authors contributed equally to this work.

***Correspondence**

Prof. Yan Jin and Dr. Cheng-Hu Hu, State Key Laboratory of Military Stomatology, Center for Tissue Engineering, Fourth Military Medical University, No. 145 West Changle Road, Xi’an, Shaanxi 710032, China. *E-mail:* yanjin@fmmu.edu.cn (Prof. Yan Jin), lshchoo@qq.com (Dr. Cheng-Hu Hu); *Tel:* +86-029-84776472; *Fax:* +86-029-83218039.

**Journal:** *Aging Cell*.

**Supporting Information:** 7 Supporting Information Figures and 1 Supporting Information Table.

**SUPPORTING INFORMATION FIGURES AND FIGURE LEGENDS**

**
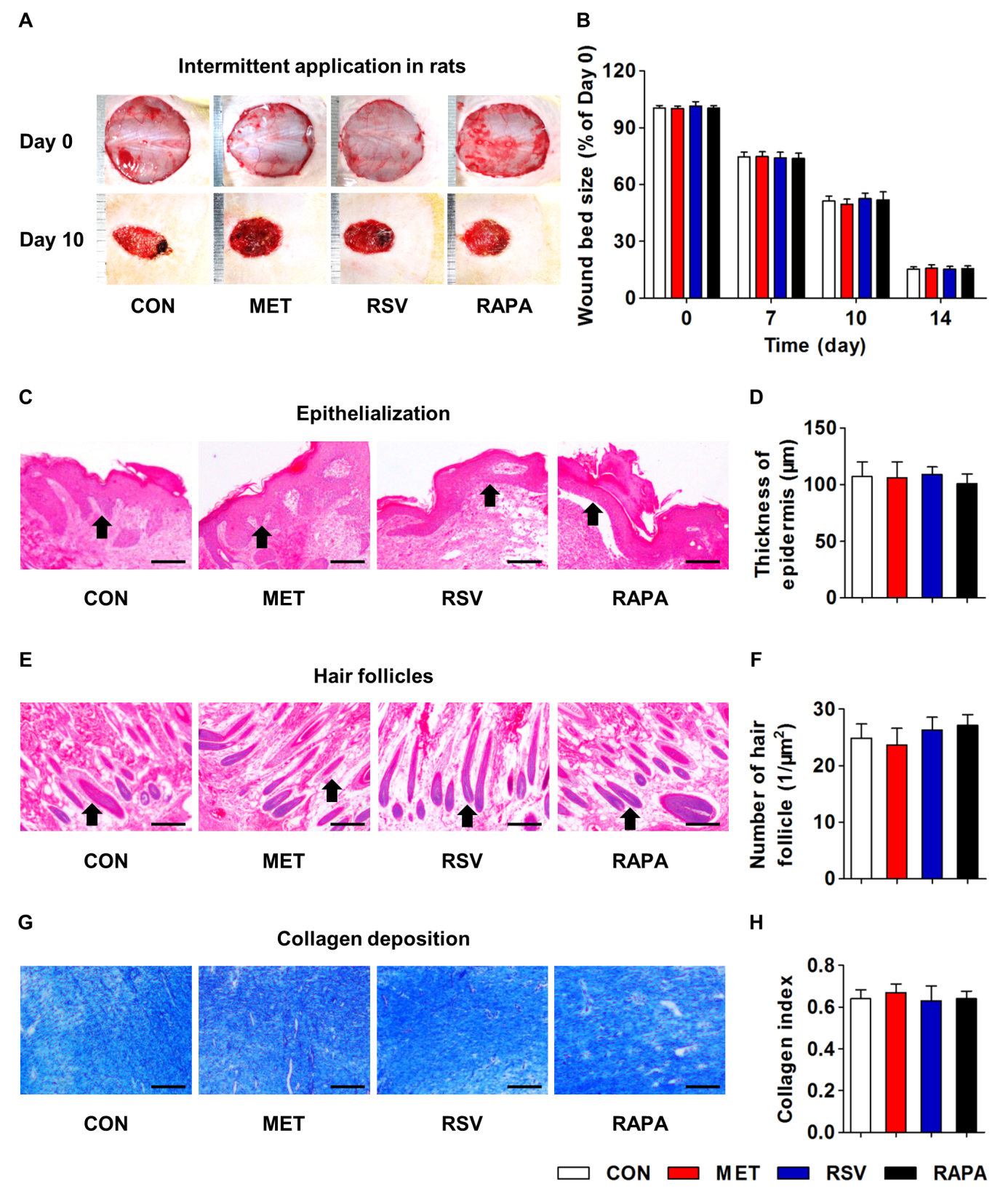
**

**Fig. S1** Cutaneous wound healing in rats with intermittent application of MET, RSV and RAPA. (A) Wound bed sizes at indicated time points in 12-week-old young rats with intermittent application of MET, RSV and RAPA and the dilution control (CON). MET, RSV and RAPA were respectively diluted at 2 μM, 50 μM and 200 nM in ethanol and applied onto the wound beds every other day for 3 times during one week followed by a treatment-free week. One scale of the ruler indicates 1 mm. (B) Quantification of wound bed sizes. (C) HE staining of wound bed samples at Day 14 showing epithelialization with black arrows indicating. (D) Quantification of thickness of epidermis. (E) HE staining of wound bed samples at Day 14 showing skin appendages of hair follicles with black arrows indicating. (F) Quantification of number of hair follicle. (G) Masson’s trichrome staining of dermal layer at Day 14 showing collagen deposition. (H) Quantification of collagen index. Bars: 100 μm. *n* = 6 per group. Data represents mean ± SD. **P* < 0.05.

**
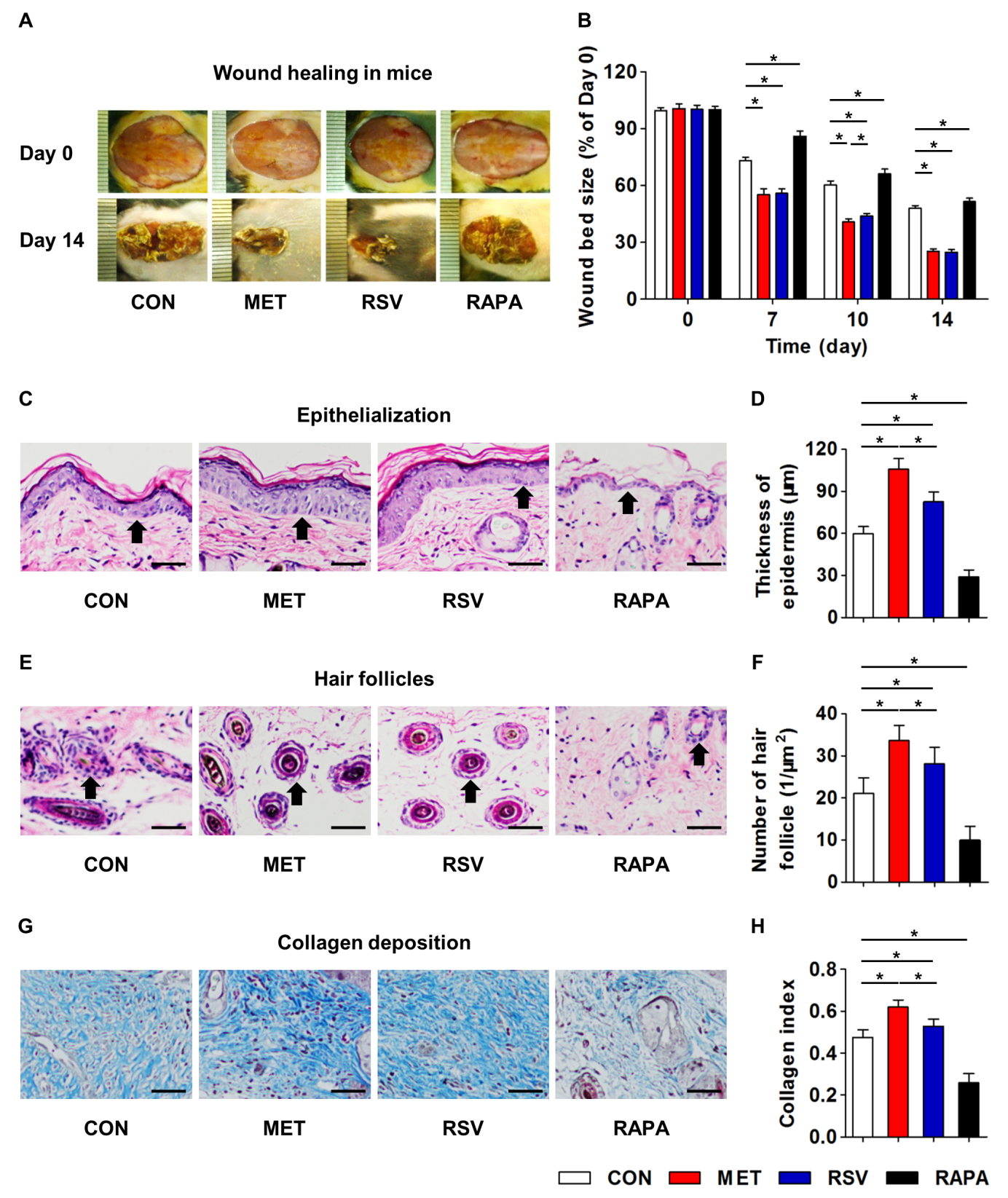
**

**Fig. S2** Cutaneous wound healing in mice with locally applied MET, RSV and RAPA. (A) Wound bed sizes at indicated time points in 12-week-old mice with locally applied MET, RSV and RAPA and the dilution control (CON). MET, RSV and RAPA were respectively diluted at 2 μM, 50 μM and 200 nM in ethanol and applied daily onto the wound beds. One scale of the ruler indicates 1 mm. (B) Quantification of wound bed sizes. (C) HE staining of wound bed samples at Day 14 showing epithelialization with black arrows indicating. (D) Quantification of thickness of epidermis. (E) HE staining of wound bed samples at Day 14 showing skin appendages of hair follicles with black arrows indicating. (F) Quantification of number of hair follicle. (G) Masson’s trichrome staining of dermal layer at Day 14 showing collagen deposition. (H) Quantification of collagen index. Bars: 100 μm. *n* = 6 per group. Data represents mean ± SD. **P* < 0.05.

**
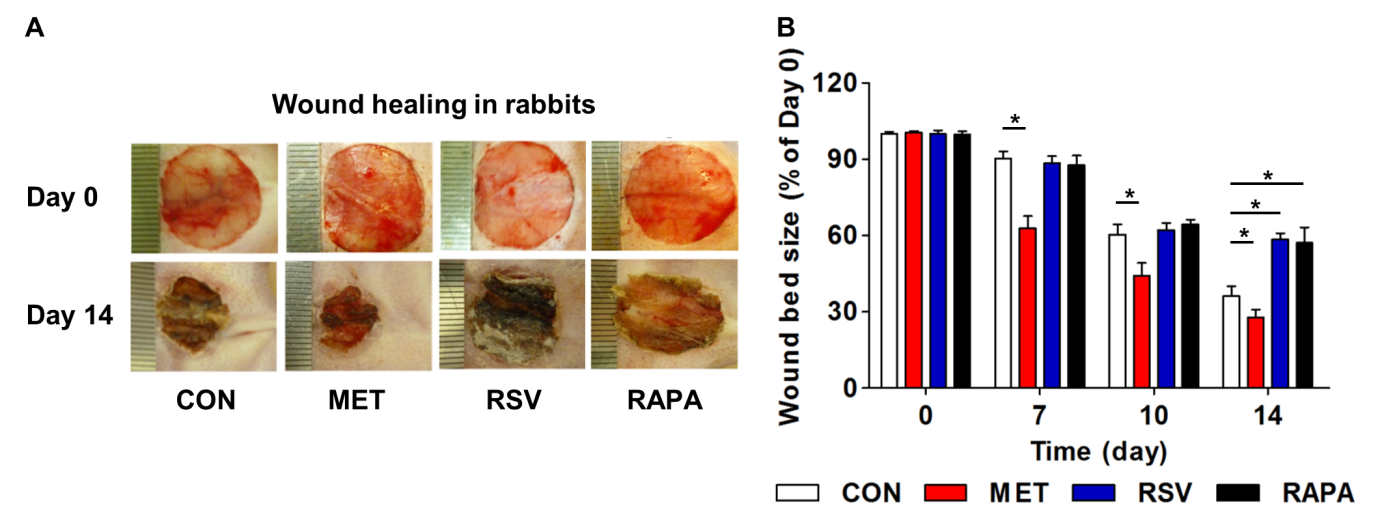
**

**Fig. S3** Full-layer cutaneous wound healing in rabbits with locally applied MET, RSV and RAPA. (A) Wound bed sizes at indicated time points in 6-month-old rabbits with locally applied MET, RSV and RAPA and the dilution control (CON). MET, RSV and RAPA were respectively diluted at 2 μM, 50 μM and 200 nM in ethanol and applied daily onto the wound beds. One scale of the ruler indicates 1 mm. (B) Quantification of wound bed sizes. *n* = 4 per group. Data represents mean ± SD. **P* < 0.05.

**
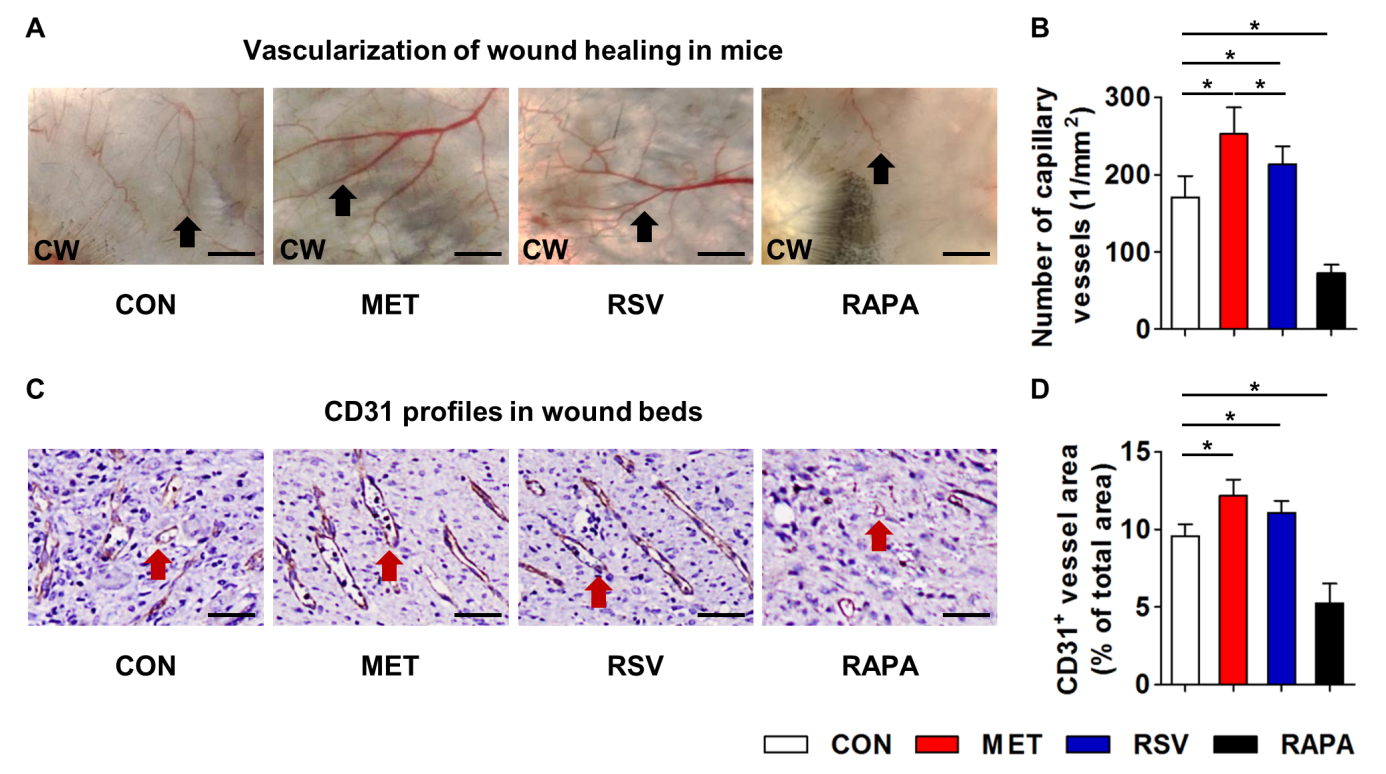
**

**Fig. S4** Vascularization of the healing wounds in mice with locally applied MET, RSV and RAPA. (A) Vascularization states of cutaneous wound (CW) beds at Day 14 with black arrows indicating capillary vessels. (B) Quantification of number of capillary vessels. (C) Immunohistochemistry staining of CD31 expression in wound bed samples with red arrows indicating the positively stained cells. (D) Quantification of percentages of CD31^+^ vessel area. Bars: 100 μm. *n* = 6 per group. Data represents mean ± SD. **P* < 0.05.


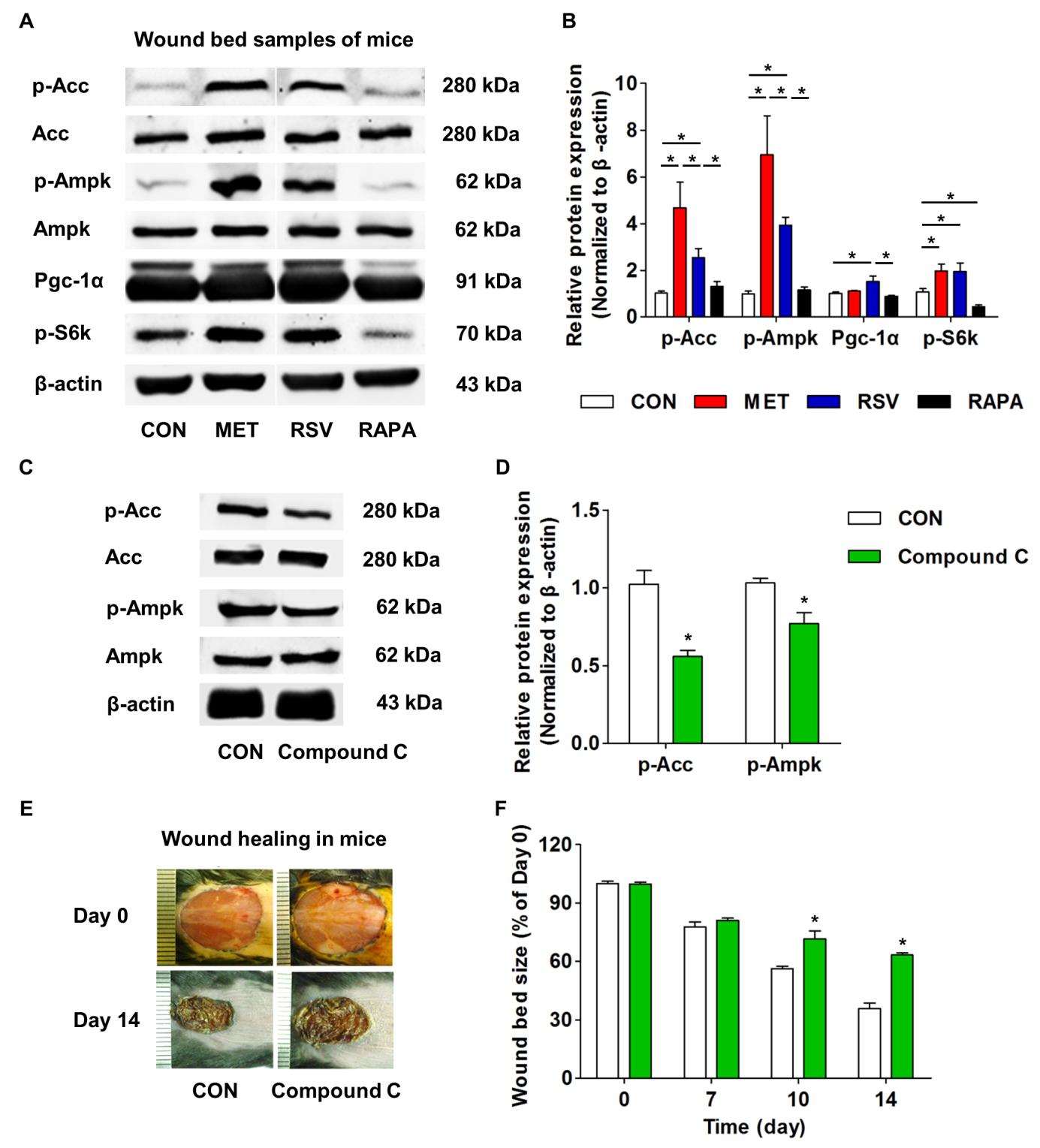


**Fig. S5** AMPK pathway plays key roles in promoting wound healing. (A) Western blot analysis on molecules of AMPK pathway (p-Acc, Acc, p-Ampk and Ampk), Sirt1 pathway (Pgc-1α) and mTOR pathway (p-S6k) in wound bed samples at Day 14 in mice with locally applied MET, RSV and RAPA. (B) Quantification of western blot data. (C) Western blot analysis on molecules of AMPK pathway in wound bed samples at Day 14 in mice with locally applied Compound C and the dilution control (CON). (D) Quantification of western blot data. (E) Wound bed sizes at indicated time points. One scale of the ruler indicates 1 mm. (F) Quantification of wound bed sizes. The AMPK inhibitor Compound C was diluted at 10 μM in ethanol and applied onto the wound beds for 100 μl/time, 1 time/d. *n* = 3 per group (B, D) and *n* = 4 per group (F). Data represents mean ± SD. **P* < 0.05.


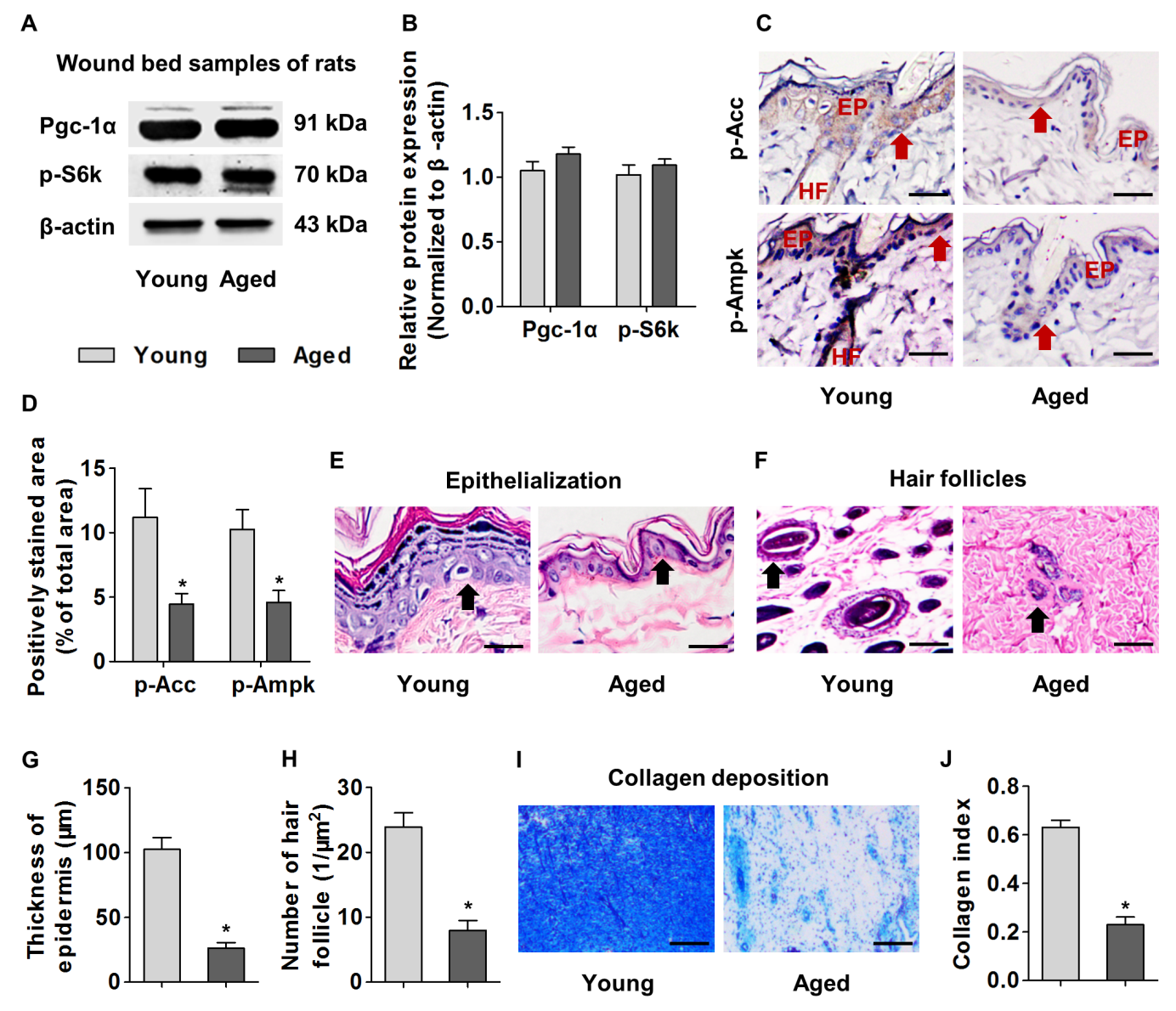


**Fig. S6** Impaired wound healing ability with inhibited AMPK signaling pathway in aged skin. (A) Western blot analysis on molecules of Sirt1 pathway (Pgc-1α) and mTOR pathway (p-S6k) in wound bed samples at Day 16 in 12-week-old (Young) and 18-month-old (Aged) rats. (B) Quantification of western blot data. (C) Immunohistochemistry staining of p-Acc (up) and p-Ampk (down) expression in wound bed samples at Day 16, with red arrows indicating the positively stained area along the epidermis (EP) and around hair follicles (HF). (D) Quantification of percentages of positively stained area. (E, F) HE staining of wound bed samples at Day 16 showing epithelialization (E) and skin appendages of hair follicles (F) with black arrows indicating. (G, H) Quantification of thickness of epidermis (G) and number of hair follicles (H). (I) Masson’s trichrome staining of dermal layer at Day 16 showing collagen deposition. (J) Quantification of collagen index. Bars: 100 μm. *n* = 3 per group (B) and *n* = 6 per group (D, G, H, J). Data represents mean ± SD. **P* < 0.05.


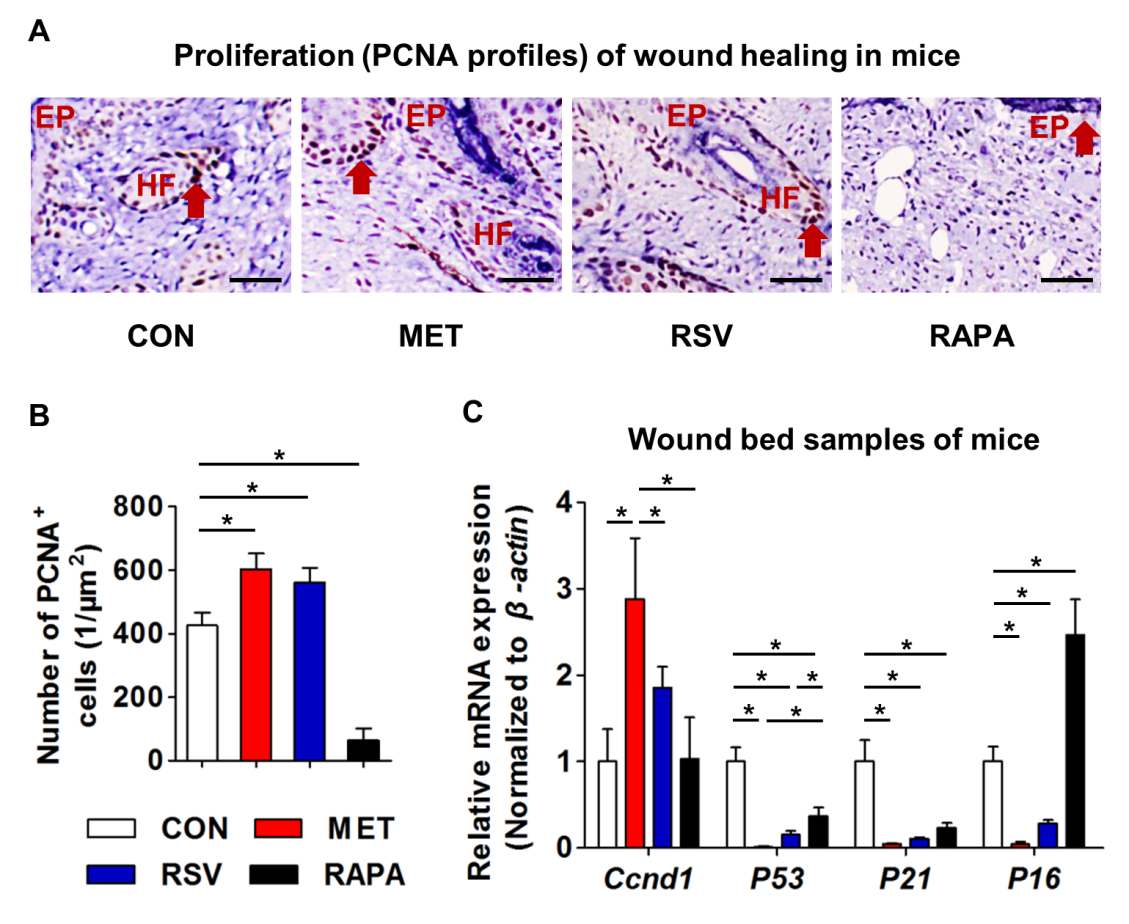


**Fig. S7** Anti-aging effects of locally applied MET, RSV and RAPA during wound healing in mice. (A) Immunohistochemistry staining of PCNA expression in wound bed samples at Day 14 with red arrows indicating the positively stained cells along the epidermis (EP) and around hair follicles (HF). (B) Quantification of number of PCNA^+^ cells. (C) qRT-PCR analysis on mRNA expression levels of the proliferative marker *Ccnd1* and senescent markers *P53*, *P21* and *P16* in wound bed samples at Day 14 in mice. Bars: 100 μm. *n* = 6 per group (B) and *n* = 3 per group (C). Data represents mean ± SD. **P* < 0.05.

**SUPPORTING INFORMATION TABLE**

**Table S1** Primer sequences in the present study for mice and rats.

| Gene | Primer sequences |
| --- | --- |
| *m-β-actin* | Forward: 5’-CATCCGTAAAGACCTCTATGCCAAC-3’  Reverse: 5’-ATGGAGCCACCGATCCACA-3’ |
| *m-Ccnd1* | Forward: 5’-AGGCGGATGAGAACAAGCAG-3’  Reverse: 5’-CCTTGTTTAGCCAGAGGCCG-3’ |
| *m-P53* | Forward: 5’-ATGAACCGCCGACCTATCC-3’  Reverse: 5’-GGCAGGCACAAACACGAAC-3’ |
| *m-P21* | Forward: 5’-CCGAAAACGGAGGCAGACC-3’  Reverse: 5’-CCGAAGATGGGGAAGAGGC-3’ |
| *m-P16* | Forward: 5’-CCAGACCGACGGGCATAG-3’  Reverse: 5’-CGCCTTCGCTCAGTTTCTCA-3’ |
| *r-β-actin* | Forward: 5’-ATGCAGAAGGAGATCACCGC-3’  Reverse: 5’-ACTCCTGCTTGCTGATCCAC-3’ |
| *r-Ccnd1* | Forward: 5’-AACTTCCTCTCCTGCTACCG-3’  Reverse: 5’-GAGATGGAGGGAGTCCTTGTT-3’ |
| *r-P53* | Forward: 5’-CAGATTGGGGAATGGGTTGG-3’  Reverse: 5’-GCAGAGTGGAGGAAATGGGTC-3’ |
| *r-P21* | Forward: 5’-CACAGGAGCAAAGTATGCCGTC-3’  Reverse: 5’-GCGAAGTCAAAGTTCCACCGT-3’ |
| *r-P16* | Forward: 5’-TCGTGCGGTATTTGCGGTAT-3’  Reverse: 5’-CCAGAAGTGAAGCCAAGGAGAA-3’ |
